# Development of a Novel Ex Vivo Porcine Hepatic Segmental Perfusion Proof-of-Concept Model Towards More Ethical Translational Research

**DOI:** 10.7759/cureus.35143

**Published:** 2023-02-18

**Authors:** Trisha Kanani, John Isherwood, Kareem ElSamani, Wen Y Chung, Kevin West, Marco R Oggioni, Giuseppe Garcea, Ashley Dennison

**Affiliations:** 1 Hepatobiliary and Pancreatic Surgery, University Hospitals of Leicester NHS (National Health Services) Trust, Leicester, GBR; 2 Histopathology, University Hospitals of Leicester NHS (National Health Services) Trust, Leicester, GBR; 3 Genetics and Genome Biology, University of Leicester, Leicester, GBR

**Keywords:** extra-corporeal, hepatic, perfusion, liver, ex-vivo

## Abstract

Introduction

Ex vivo machine perfusion describes the technique where organs are continuously perfused and oxygenated extracorporeally (at physiological conditions) to maintain the organs' viability. To our knowledge, there are currently no reported studies describing ex vivo perfusion of a single hepatic segment. Here, we describe the development of a porcine ex vivo hepatic segmental perfusion model to demonstrate proof of concept and support further research into the ex vivo perfusion of the human liver using discarded tissue.

Methods

Whole livers were retrieved from abattoir-derived pigs and connected to a normothermic extracorporeal perfusion circuit. Constant segmental perfusion via the common or segmental hepatic artery and portal vein with heparinised autologous blood was established. The viability of the perfused organ was assessed by monitoring perfusion pressures, flow rates and histology samples.

Results

Following perfusion and optimisation of the model for three hepatic segments, the third perfusion demonstrated viable hepatocytes centrally after 4 h of segmental perfusion.

Conclusion

Ex vivo hepatic segmental perfusion is technically challenging but its success in a porcine model and the principles learned should facilitate the development of an analogous human model using discarded tissue following formal liver resections. The model would use a healthy liver segment following a major formal resection such as a hemi-hepatectomy and ex vivo perfusion performed via a segmental hepatic artery and portal vein. If successful this model would represent a significant development and enable ethical translation research to assess the response of human livers to a variety of stressors, including toxicity and infection.

## Introduction

Ex vivo machine perfusion describes a technique in which organs are continuously perfused and kept oxygenated extracorporeally under physiological conditions to maintain an organ's viability.

Ex vivo perfusion of porcine organs has proved to be a valuable tool in translational research due to the remarkable similarities between human and porcine anatomy and physiology. These porcine organ perfusion models have been employed for the study of organ transplantation and the development of ex vivo perfusion technology but have also been used widely to study invasive diseases [1]. Wanford et al. used ex vivo perfusion models of porcine liver and spleen to demonstrate intracellular replication of *Klebsiella pneumoniae* in tissue macrophages, highlighting the pathophysiology of hepatic abscess formation and the importance of optimising antimicrobial treatment strategies [2]. These organ perfusion models confer significant economic benefits and importantly are an ethical alternative to live animal experiments.

Russel and Burch described the “3Rs” of animal research in 1959, which are replacement, reduction and refinement [3]. Ex vivo porcine hepatic perfusion provides important data and for many studies is a successful alternative to live animal experiments. Ex vivo perfusion of human livers would completely abrogate the need to sacrifice animals, provide a more physiological model to study hepatic disease and furnish the essential human data which are frequently essential prior to safe clinical trials. Furthermore, there is an inexorable increase in the demand for donor livers for transplantation and the introduction of successful ex vivo perfusion for preservation and recovery of marginal donor livers reduces the availability of discarded human livers which have previously been an important source of organs for research.

In an effort to increase the availability of transplantable human liver grafts, split-liver perfusion of the left and right hepatic lobes has also been reported [4]. Schreiter et al. described a human ex vivo model for acetaminophen-induced liver injury [5]. This study describes the perfusion of healthy human tissue retrieved from the pathology department following partial hepatectomy with perfusion performed via a single inflow vessel which subsequently divided into four. A limitation of such perfusion is that it does not reflect anatomical liver perfusion via the portal vein (PV) and hepatic artery (HA). We propose that ex vivo perfusion of a liver segment should allow for a single segmental branch of the HA and PV to be cannulated with individual control of their perfusion parameters.

To our knowledge, there are currently no reported studies describing ex vivo perfusion of an isolated hepatic segment [6]. The development of a novel ex vivo human liver segmental perfusion model would address the three principles of replacement, reduction and refinement and provide a versatile model for a wide range of pre-clinical studies [3]. We describe the development of a porcine ex vivo hepatic segmental perfusion model to demonstrate proof of concept and support further research into the ex vivo perfusion of human liver segments. This is an initial report, preliminary to the development of an ex vivo perfusion model for isolated human liver segments. 

## Materials and methods

We describe the ex vivo perfusion of the porcine hepatic segment (PHS) 1, PHS2 and PHS3 during the development of this feasibility model. 

Animal procurement

Livers were retrieved from large white pigs sourced from a Foods Standards Agency-approved abattoir (Joseph Morris Butchers Ltd, South Kilworth, Leicester, UK). Pigs were slaughtered as part of the routine abattoir process in accordance with UK legislation. Organ retrieval for this study did not interfere with meat production. 

Following terminal exsanguination from the carotid artery and jugular vein, 1500 ml of autologous blood was retrieved in non-sterile conditions in an autoclaved, pre-heparinised container holding 25,000 units of unfractionated heparin. This was designated the beginning of the warm ischaemic (WI) time. 

Liver retrieval 

Retrieval of the porcine livers represented a donation after cardiac death (DCD) model. Once there was no sign of life, the pig was positioned supine on a retrieval table and a full midline thoraco-laparotomy was performed to gain access to the thorax and abdomen. 

Following the sharp dissection of the diaphragm and pleurae, the suprahepatic inferior vena cava (IVC) was divided. The small intestine and transverse colon were retracted caudally to expose the duodenum. The third part of the duodenum was divided. The pancreas and duodenum were lifted cranially to expose the junction of the superior mesenteric and splenic veins and both vessels were ligated. The splenic artery was identified and traced down to its origin from the coeliac trunk. Once the origin of the coeliac trunk was identified at the aorta, the HA was cannulated in vivo with a 3.3 mm female catheter (Pennine Healthcare®, Derby, UK)and secured with Ethicon® Coated Vicryl^TM ^(polyglactin 910) ties in size 0 (Johnson & Johnson Medical NV, Machelen, Belgium). The common bile duct and left gastric, splenic and gastroduodenal arteries were identified and ligated with 0 Vicryl^TM^ ties. The junction between the pylorus and duodenum was identified and divided and the infrahepatic IVC was identified and divided.

**Figure 1:**
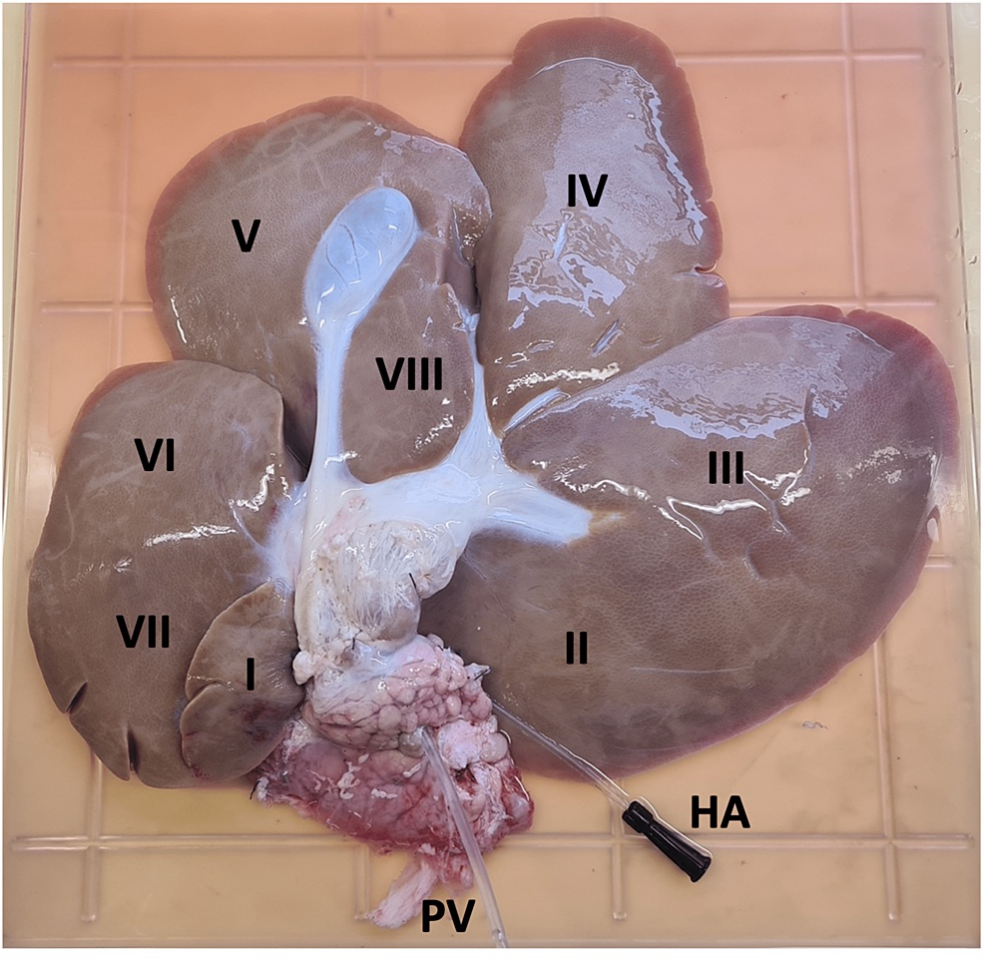
Segmental anatomy of the porcine liver; posterior view with cannulated common hepatic artery (HA) and portal vein (PV).

All hepatic peritoneal and visceral attachments were divided to mobilise the liver. Once free, the liver was retrieved and immediately placed on ice. The PV was cannulated on the back table with a 4.7 mm nelaton catheter (Pennine Healthcare®, Derby, UK) and secured with 0 Vicryl^TM^ ties. The PV and HA were each flushed with 1000 ml ice-cold normal saline containing 25,000 units of unfractionated heparin. This was designated the end of the WI time and the beginning of the cold ischaemic (CI) time. Following infusion with heparinised saline, 1000 ml ice-cold Soltran® Kidney Perfusion Solution (Baxter Healthcare Ltd, Norfolk, England) or Custodiol® HTK Solution (Dr. Franz Köhler Chemie GmbH, Bensheim, Germany) was administered through each vessel. The liver was then transported on ice in an Invisishield^TM^ isolation bag (Medline International, France), containing a preservation solution, to the laboratory. Figure 1 demonstrates the retrieved porcine liver and its segmental anatomy.

Ex vivo perfusion circuit

Extracorporeal perfusion was achieved with a bespoke custom-designed perfusion set supplied by the Cardiac Surgery Division (The Netherlands, Europe) of Medtronic Inc. (Minneapolis, MN, USA). The set provided included an AFFINITY PIXIE^TM^ Hollow Fiber Oxygenator with Balance Biosurface and AFFINITY PIXIE^TM^ Cardiotomy/Venous Reservoir with Balance^TM^ Biosurface and a portal venous reservoir bag. 

The circuit was assembled and connected to a Bio-Console 560^TM^ Pump Speed Controller (Medtronic Inc, Minneapolis, MN, USA) and an AFFINITY^TM^ CP centrifugal pump motor (Medtronic Inc, Minneapolis, MN, USA) set to a speed of 2000 rpm. This system allowed continuous pressure and flow monitoring for the PV and HA. The temperature was controlled with a GD120 series water bath by Grant Instruments Ltd (Cambridge, UK) set to 39°C and 100% oxygen was supplied from a gas cylinder with a flow rate of 1 l/min. 

**Figure 2:**
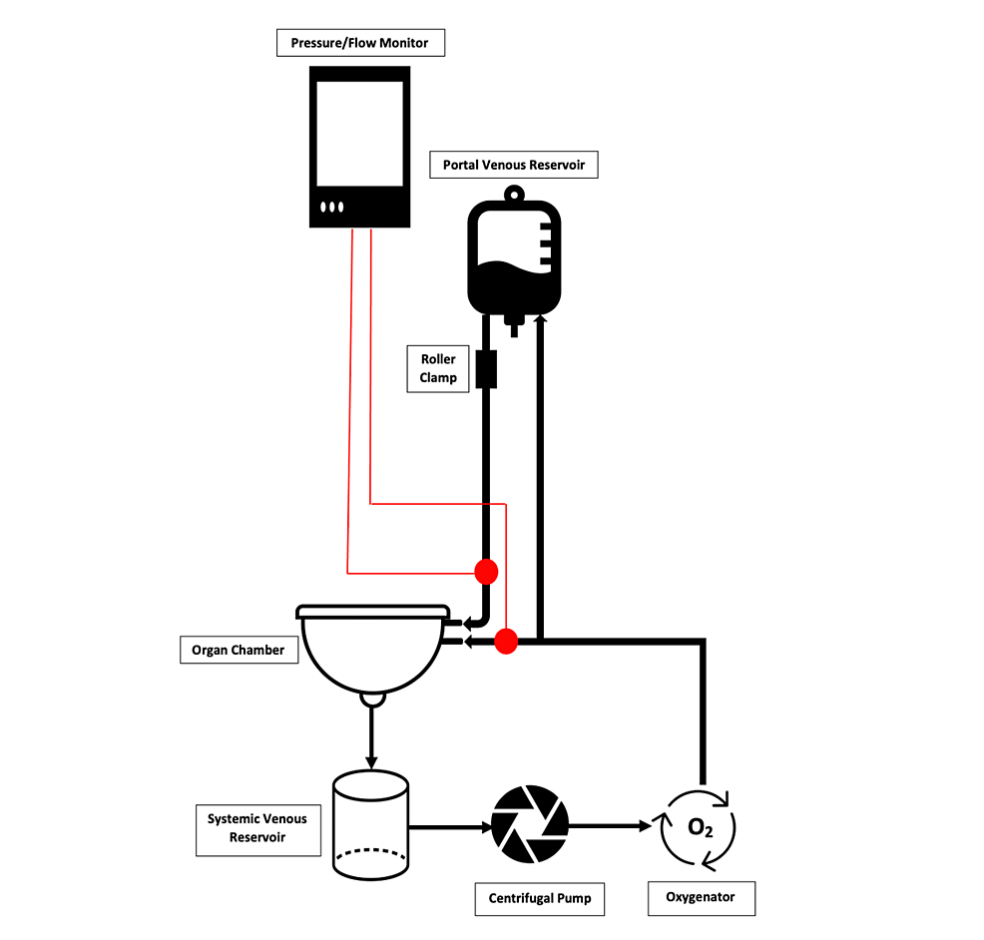
Schematic diagram of the ex vivo perfusion circuit.

Figure 2 shows a schematic diagram of the ex vivo perfusion circuit. Hepatic venous blood is collected in the organ chamber and drains into the systemic venous reservoir. From there, it enters the centrifugal pump to generate a higher vascular pressure and then passes through the oxygenator. The high-pressure oxygenated blood is split with a Y-connector with one branch going directly to the HA and one diverted to the portal venous reservoir. From the portal venous reservoir, blood flows by gravity to the PV at a lower pressure controlled with a roller clamp. 

Hepatic segment isolation

**Figure 3:**
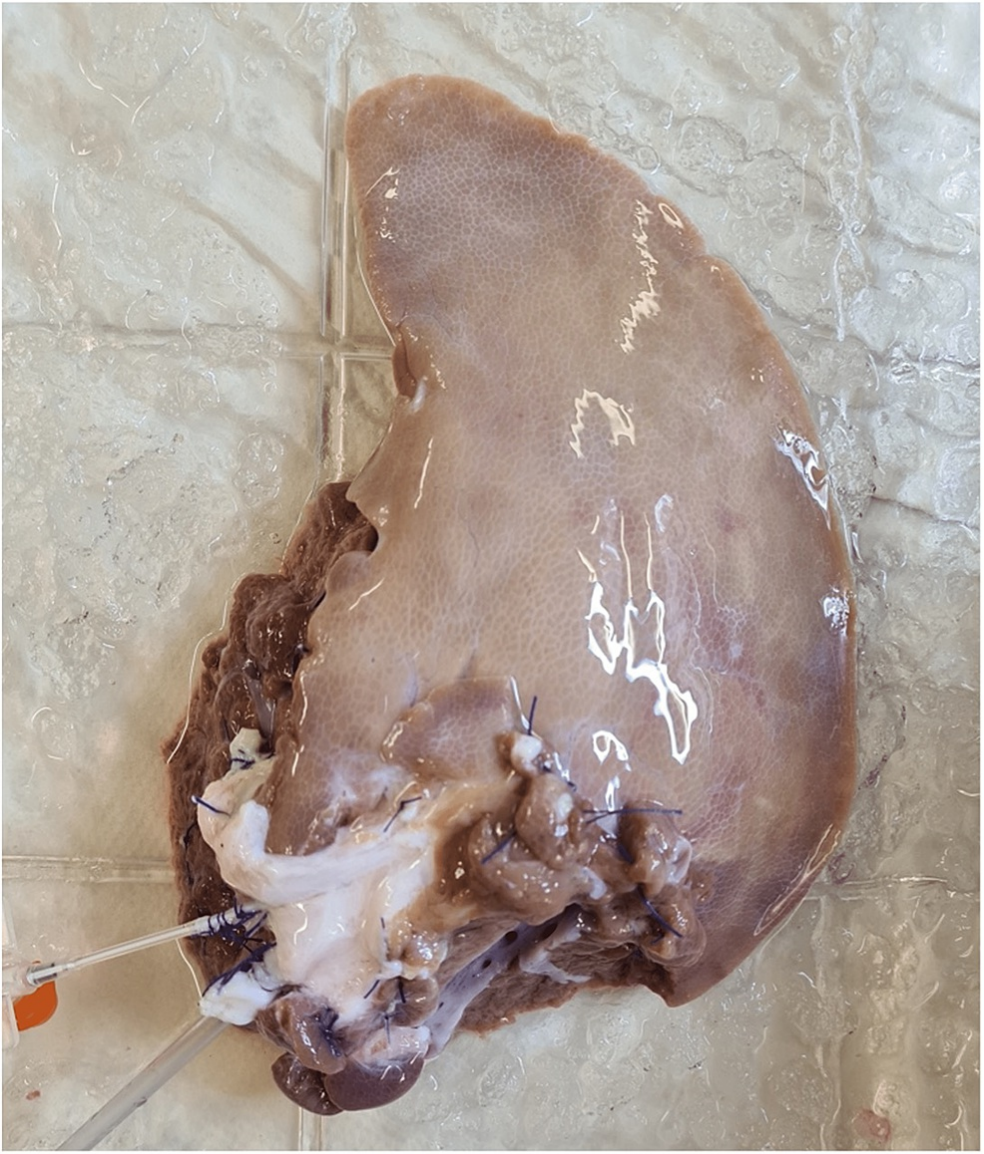
Porcine hepatic segment 1; segment IV with cannulated segmental hepatic artery and portal vein prior to perfusion.

PHS1 perfusion was achieved by performing an ex vivo backtable right hemihepatectomy and left lateral lobectomy with 2/0 Vicryl^TM^ ties and Ethicon® 5-0 Prolene^TM^ sutures (Johnson & Johnson Medical NV, Machelen, Belgium), to isolate segment IV. The PV and HA were dissected from the hilum to the branches to segment IV. The segmental PV branch was cannulated with a 4.7 mm nelaton catheter and the segmental HA was cannulated with a 14G intravenous catheter (Figure 3). 

For PHS2 and PHS3, liver resection was performed during ex vivo perfusion of the whole liver to leave just segment IV and segment VI perfused, both from the common HA and PV as opposed to the segmental branches described for PHS1. All three segments were perfused for 4 h. 

Optimising perfusate 

As described above, 1500 ml of autologous blood from the porcine carotid artery and jugular vein was collected in a sterile heparinised container. Immediately prior to perfusion, boluses of drugs as outlined in Table 1 were administered to the whole blood. Drugs were also administered as infusions into the systemic venous reservoir (Table 1). Soltran® was the preservation solution used for PHS1 and PHS2. Due to a limited supply, Custodiol® preservation solution was used for PHS3. The perfusate was further optimised for PHS3 with the addition of magnesium sulphate, dexamethasone, N-acetyl-cysteine (NAC) boluses and an insulin infusion.

**Table 1:** Drugs administered to optimise perfusate

Segment	Preservation Solution	Drug Boluses	Infusions
PHS1 Segment IV	Soltran®	25000 units heparin 750 mg cefuroxime 2 mmol calcium chloride 5 ml 8.4% sodium bicarbonate 50 µg epoprostenol sodium	500 µg epoprostenol sodium in 200 ml normal saline (20 ml/h) 25000 units heparin in 250 ml normal saline (40 ml/h)
PHS2 Segment IV	Soltran®	25000 units heparin 750 mg cefuroxime 5 mmol calcium chloride 20 ml 8.4% sodium bicarbonate 50 µg epoprostenol sodium	500 µg epoprostenol sodium in 200 ml normal saline (20 ml/h) 25000 units heparin in 250 ml normal saline (40 ml/h)
PHS3 Segment VI	Custodiol®	25000 units heparin 750 mg cefuroxime 5 mmol calcium chloride 25 ml 8.4% sodium bicarbonate 50 µg epoprostenol sodium 1 g magnesium sulphate 3.3 mg dexamethasone 400 mg NAC	500 µg epoprostenol sodium in 200 ml normal saline (20 ml/h) 25000 units heparin in 250 ml normal saline (40 ml/h) 200 units insulin in 250 ml normal saline (10 ml/h)

Data collection

Perfusion parameters were displayed continuously on the Bioconsole 560 monitor with a pressure and flow recording of the blood supplying both the HA and PV. Blood gases were taken at 10 min, 1 h, 2 h and 4 h of perfusion to establish changes in lactate levels. Liver enzymes were not measured as the primary objective in the porcine model was to establish histological viability and marked hepatocellular injury would be expected in an abattoir-derived porcine liver segmental model. Excision biopsies were taken before and after resection and following 2 and 4 h of segmental perfusion. Biopsy specimens were transported to the histopathology department in 10% formalin and underwent haematoxylin and eosin (H&E) staining of paraffin-embedded sections. Histology was reviewed with light microscopy and reported by a Consultant Histopathologist. 

## Results

The ischaemic times and perfusion parameters for all three segments are displayed in Table 2. 

**Table 2:** Ischaemia times and perfusion parameters for porcine hepatic segments 1, 2 and 3 WI, warm ischaemic; CI, cold ischaemic; PV, portal vein; HA, hepatic artery; PHS, porcine hepatic segment.

Segment	WI time (minutes)	CI time (minutes)	Mean PV flow (l/min)	Mean PV pressure (mmHg)	Mean HA flow (l/min)	Mean HA pressure (mmHg)
PHS1	28	270	0.22	8.5	0.04	81
PHS2	24	165	0.20	9	0.17	84
PHS3	42	150	0.36	11	0.14	78

**Figure 4:**
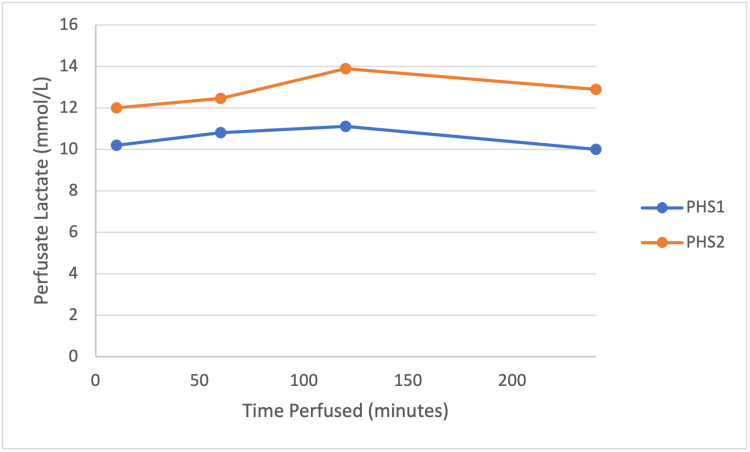
Lactate levels during perfusion for PHS1 and PHS2. PHS, porcine hepatic segment.

Figure 4 shows lactate trends on blood gases for PHS1 and PHS2. PHS3 did not give recordable lactate results and is therefore excluded from the graph. 

PHS1 histology demonstrated normal histology prior to perfusion, minor haemorrhage and peripheral degenerative changes at 2 h of perfusion and at 4 h of perfusion demonstrated widespread haemorrhage throughout the liver with peripheral degenerative changes in hepatocytes.

PHS2 showed early degeneration of hepatocytes with the whole liver perfused with no haemorrhage. Following resection, with only segment IV perfused, there was sinusoidal dilatation, haemorrhage and focal degeneration of hepatocytes. After 2 h of perfusion, there was a significant haemorrhage suggested of sinusoidal degeneration. The 4-hour sample demonstrated widespread haemorrhage throughout the liver with peripheral degenerative changes in hepatocytes, similar to PHS1. 

PHS3 demonstrates normal histology at 2 h of perfusion and that the majority of hepatocytes remain viable with only subcapsular degeneration at 4 h, despite unrecordable lactate levels. 

**Figure 5:**
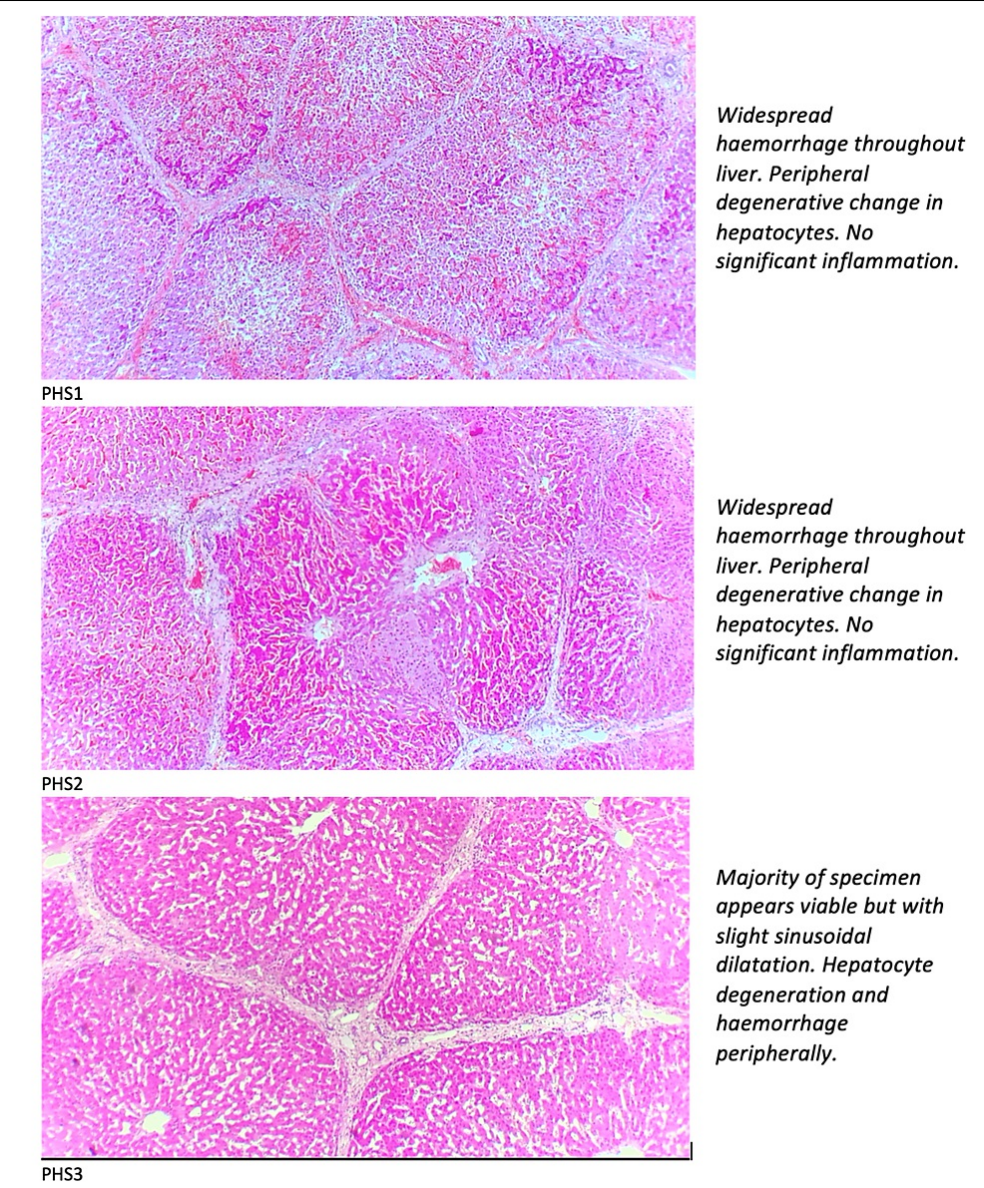
H&E staining for porcine hepatic segment 1, 2 and 3 at 4 h of ex vivo perfusion and report from the Consultant Histopathologist. H&E, haematoxylin and eosin.

Figure 5 shows the H&E-stained biopsies magnified under a light microscope and the report from a Consultant Histopathologist for the excision biopsies taken at 4 h of segmental perfusion. 

## Discussion

PHS1 and PHS2 were both segment IV perfusions and underwent different resection techniques with PHS1 resected on ice and PHS2 resected during ex vivo perfusion of the whole liver. Soltran® preservation solution was used for both perfusions and drug boluses and the infusions were identical. As PHS1 perfusion involved cannulation and perfusion via segmental branches of the HA and PV, this would be most similar and translatable to ex vivo human liver segmental perfusion following surgical hepatectomy. 

This study used porcine organs retrieved from an abattoir and therefore the livers had prolonged WI times, with a marked degree of ischaemia reperfusion injury expected [7]. For this reason, viability was assessed using histological analysis and stability of lactate levels for these preliminary studies. Liver enzymes were not measured due to the expected and unreliable derangements following prolonged WI times in an abattoir-derived model undergoing resection during or prior to perfusion. Results above show that at 4 h of segmental perfusion, biopsies from both PHS1 and PHS2 demonstrated widespread haemorrhage throughout the liver. These histological changes are in keeping with gross degeneration of liver architecture including the sinusoids which cause haemorrhage into the tissue [8]. 

During the development of this model, several changes were made to PHS3. A different preservation solution (Custodiol® ) was used due to supply issues. Moreover, boluses of magnesium sulphate, NAC and dexamethasone were given in addition to the boluses administered to PHS1 and PHS2 (Table 1). As shown in Figure 5, the 4-hour biopsy results were promising with viable hepatocytes centrally reflecting successful perfusion and peripheral degeneration and mild sinusoidal dilatation may be a result of ischaemia reperfusion injury or high perfusion pressures for the HA [9]. The changes to drugs administered to PHS3 were adopted following a discussion with a tertiary transplant centre perfusion department who added this combination of drugs to their livers perfused ex vivo with OrganOx (OrganOx Ltd, Oxford, UK). The anti-inflammatory and antioxidant properties of these drugs are likely to have improved the histological outcomes [10].

We demonstrate here the first proof of concept for successful ex vivo perfusion of single liver segments using a porcine model. There are limitations to this study including the prolonged WI time for the abattoir-derived retrievals. These limitations are acceptable to the study group given that this model allowed ethical research to be performed where porcine organs would otherwise have been discarded at an abattoir and no additional pigs were sacrificed for the study. 

Furthermore, liver enzymes were not monitored as a marker of injury as it would be anticipated that there would be gross derangement following retrieval of an abattoir-derived liver which underwent resection prior to, or during, perfusion. Therefore, optimising the model to demonstrate histological viability was deemed to be sufficient to provide a feasibility model for human liver segmental perfusion. 

Ex vivo hepatic segmental perfusion is technically challenging but the demonstration of the feasibility in a single porcine model should be deemed sufficient to facilitate similar studies on human liver segments in an effort to avoid unethical animal experimentation. Hemi-hepatectomies, most commonly performed for colorectal liver metastases and hepatocellular carcinoma, are frequent procedures in high-volume tertiary hepato-pancreato-biliary units [11]. The retrieval of a healthy liver segment following these procedures and subsequent ex vivo perfusion via a segmental HA and PV will confer economic and ethical benefits and facilitate translational research based on data from human liver subjected to toxic and infective insults together with therapeutic approaches to modulate the effects. A human segmental perfusion model will require careful optimisation of vessel cannulation, WI and CI times and a suitable oxygen carrier but our study demonstrating viable hepatocytes (PHS3) after 4 h of segmental perfusion indicates that this will be achievable. 

## Conclusions

Future studies require the establishment of clinical trials to allow research use of human liver tissue following surgical resection. Surgical techniques to allow vessel cannulation and to secure the perfusion cannulae effectively would need to be established, as would the perfusate and drug optimisation. Another variable to consider with human liver segmental perfusion is that the time of WI would begin during surgical resection when the inflow to the resected specimen is first ligated. 

The exciting development of such a novel model would allow more translational experimentation on perfused human liver tissue, which would be representative of the organ and would avoid animal experimentation. 
